# A scoring system for screening of patients with synchronous bone metastasis in lung cancer

**DOI:** 10.1097/MD.0000000000046889

**Published:** 2026-01-09

**Authors:** Weiqin Zhao, Yile Lin, Haixiao Wu, Jun Wang, Vladimir P. Chekhonin, Andrey D. Kaprin, Elmar R. Musaev, Jin Zhang, Xu Guo, Chao Zhang

**Affiliations:** a Outpatient Department, Cangzhou Central Hospital, Cangzhou, Hebei Province, P.R. China; b The Sino-Russian Joint Research Center for Bone Metastasis in Malignant Tumor, Tianjin, China; c Moscow Oncological Hospital No. 62 of the Moscow Department of Health, Moscow, Russia; d Department of Bone and Soft Tissue Tumor, Tianjin Medical University Cancer Institute and Hospital, National Clinical Research Center for Cancer, Key Laboratory of Cancer Prevention and Therapy, Tianjin’s Clinical Research Center for Cancer, Tianjin, China; e Department of Neurology, Neurosurgery and Medical Genetics, Department of Medical Nanobiotechnology, Pirogov Russian National Research Medical University of the Ministry of Healthcare of the Russian Federation, Moscow, Russia; f P.A. Hertsen Moscow Oncology Research Center – Branch of Federal State Budgetary Institution National Medical Research Radiological Center of the Ministry of Health of the Russian Federation, Moscow, Russia; g Department of Orthopedics, Cangzhou Central Hospital, Cangzhou, Hebei Province, China.

**Keywords:** bone metastasis, lung cancer, screening

## Abstract

This study aims to investigate the incidence of and factors associated with bone metastasis (BM) in patients with lung cancer and provide guidance for the screening of BM in patients with lung cancer. Lung cancer patients who were treated at the Tianjin Medical University Cancer Institute and Hospital between 2009 and 2018 were retrospectively reviewed. Patients with synchronous BM were obtained to investigate the risk factors for BM development. Both demographic and clinical characteristics were included. Univariate and multivariate logistic regression analyses were performed to identify risk factors. A further scoring system was established to classify patients into 3 different risk groups. Five significant risk factors were identified, namely, male sex, Karnofsky score of 50 to 70, multiple primary lesions, lymph node metastasis, and adenocarcinoma. The total score was generated by the integration of the score based on each individual factor. The incidence of BM among patients with lung cancer with a total score ≥ 7 was 46.73%, the incidence among patients with a total score ≤ 4 was 16.59%, and the incidence of BM in patients with total scores between 5 and 6 was 34.23%. This scoring system can predict the risk of BM in patients with lung cancer and can guide the performance of BM screening.

## 1. Introduction

Lung cancer is one of the leading causes of cancer-related death, accounting for approximately 21% of cancer-related deaths worldwide.^[1]^ The poor prognosis of lung cancer patients is significantly related to the occurrence of distant metastasis.^[2,3]^ More than 65% of lung cancer patients present with local and/or disseminated metastatic disease at the time of diagnosis.^[4–6]^

Bones among the most common sites of lung cancer metastasis. Once bone metastasis (BM) occurs, approximately 80% of lung cancer patients suffer severe pain and poor quality of life (QOL).^[7–10]^ BM can be divided into synchronous BM (SBM) and metachronous BM (MBM) according to the diagnosis.^[11]^ There is still no consensus about the definition of SBM. To standardize the study of BM, SBM was defined as a BM diagnosis within 3 months of cancer diagnosis.^[12]^ The clinical diagnosis of BM in patients with lung cancer usually relies on patients’ clinical symptoms and skeletal imaging findings. Unfortunately, there is no effective way to detect bone destruction until the metastatic tumors are radiographically detectable.^[13]^

Over 60% of patients with BM suffer from skeletal-related events (SREs), which are defined as severe pain, surgery and/or radiotherapy to bone; pathological fractures; and spinal cord compression and/or hypercalcemia.^[14–17]^ SREs have been proven to significantly worsen the prognosis of patients.^[18]^ In a clinical trial, most patients with non-small cell lung cancer BM experienced an SRE within the 1st 5 months. Bone lesions may be overlooked because they are not diagnosed until patients present with bone pain or other signs of bone involvement.^[19]^ Importantly, the consequences of SREs may persist throughout the lifetime of these patients. Therefore, screening for BM before the onset of SREs can guide bone-targeting therapy and offer more treatment opportunities, thus potentially improving the survival and quality of life of lung cancer patients. At present, there are few clinically effective methods for predicting BM in patients with lung cancer.^[20,21]^ To further improve the level of prediction, diagnosis, and disease monitoring of BM in patients with lung cancer, a new scoring system is needed.

In this study, lung cancer patients who were treated at the Tianjin Medical University Cancer Institute and Hospital between January 2009 and December 2018 were selected. Patients with SBM were obtained to explore the clinical characteristics and risk factors that affect the development of BM in patients with lung cancer. We aimed to estimate the risk of SBM in lung cancer patients and created a scoring system for predicting BM. This system could guide the initial screening for BM in lung cancer patients and timely bone-targeting therapy.

## 2. Methods

**Figure 1.**
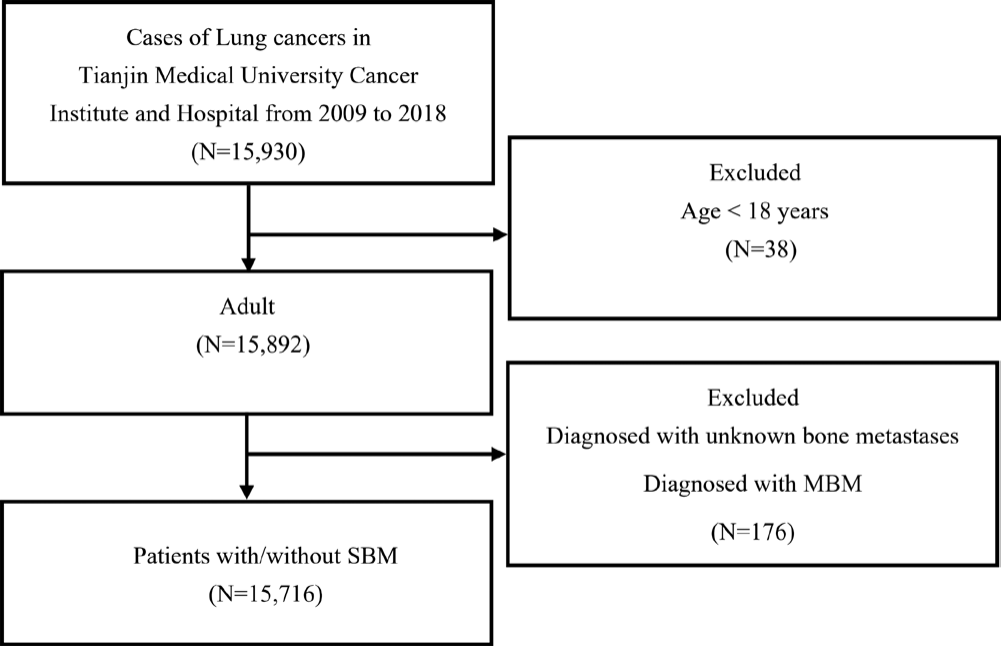
The flowchart of the patients’ selection for the morbidity of SBM in lung cancer. SBM = synchronous bone metastasis.

This retrospective analysis was approved by the Ethics Committee of Tianjin Medical University Cancer Institute & Hospital. A total of 15,930 lung cancer patients were initially diagnosed in the hospital from January 2009 to December 2018. The exclusion criteria were: patients who were younger than 18 years and patients who were diagnosed with unknown BM or MBM in lung cancer. An unknown BM was defined as an uncertain BM occurrence. SBM was defined as a BM diagnosis within 3 months of the lung cancer diagnosis, whereas MBM was defined as a BM diagnosis more than 3 months after the lung cancer diagnosis.^[12]^ A total of 15,716 lung cancer patients were ultimately included according to the exclusion criteria. The flowchart of the subject selection process is shown in Figure 1.

### 2.1. Statistical analysis

The demographic and clinical characteristics of the patients were as follows: age (18–45 years, 46–65 years or > 65 years), gender (female or male), marital status (married and other status or unmarried), history of smoking (yes or no), alcohol consumption (yes or no), history of cancer (yes or no), family history of cancer (yes or no), Karnofsky score (10–40, 50–70, or 80–100), primary lesions (1 or > 1), lymph node metastasis (yes or no), and histological type (small cell lung cancer, adenocarcinoma, squamous cell carcinoma, large cell lung cancer, mixed lung cancer or others). For each variable with missing data, we created an “Unknown” category, and these samples were analyzed as a distinct group.

Univariate and multivariate logistic regression analyses were performed to determine the risk factors for developing SBM. The results of the multivariate analyses were expressed in terms of hazard ratios derived from the estimated regression coefficients, with 95% confidence intervals. The score for each significant risk factor was derived from the corresponding estimated regression coefficients (natural logarithm of the hazard ratio) of the multivariate model. The corresponding estimated regression coefficients were multiplied by 3 and rounded to the nearest integer. In this way, the factors with the smallest regression coefficients were allocated 1 point. The final total score was generated by adding the scores of each factor. The differences in the BM incidence rates of the 3 risk groups were determined by the *χ*^2^ test. All the statistical analyses were performed using SPSS 23.0 (IBM Corporation, Armonk), and 2-sided *P* values < .05 were considered statistically significant.

## 3. Results

**Figure 2.**
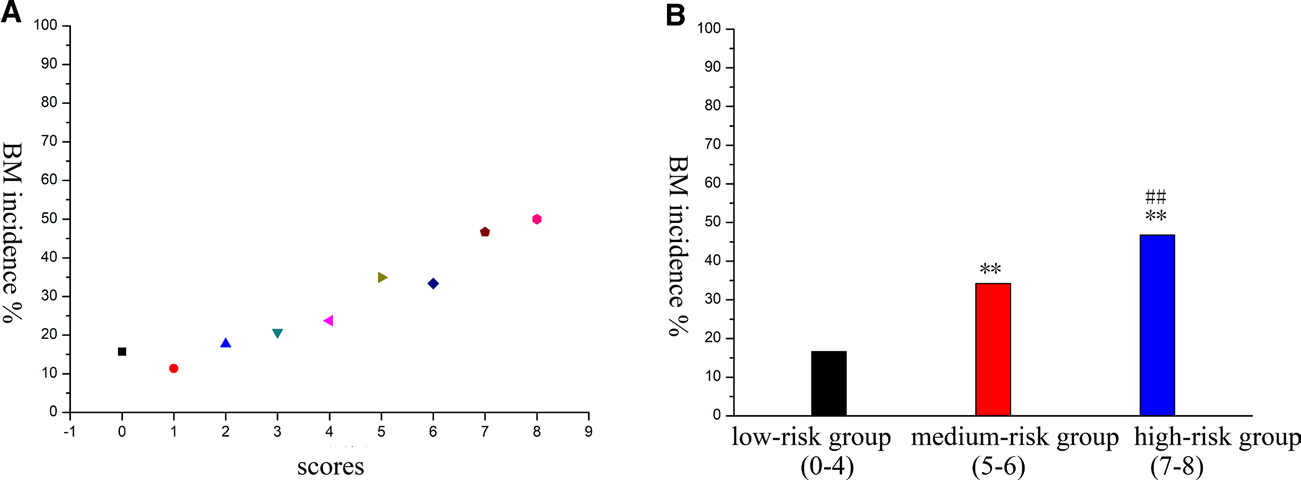
The patients were stratified into 9 distinct groups based on the generated scores, ranging from (0–8) (A). Regarding bone metastasis (BM) risk stratification (B), lung cancer patients were categorized into 3 groups as follows: patients with comparable BM incidence in the range of (0–4) were allocated to the low-risk group; those with similar BM incidence from 5 to 6 were assigned to the medium-risk group; and patients exhibiting comparable BM incidence in the range of 7 to 8 were classified into the high-risk group. BM = bone metastasis. ** *P* < .01(vs low-risk group). ## *P* < .01 (vs medium-risk group).

Among the 15,716 included lung cancer patients, 2738 (17.42%) patients were diagnosed with SBM (Table 1). Male sex, Karnofsky score of 50 to 70, multiple primary lesions, lymph node metastasis, and adenocarcinoma were found to be significant and independent risk factors for BM in patients with lung cancer. The hazard ratios for male sex, Karnofsky score of 50 to 70, multiple primary lesions, lymph node metastasis, and adenocarcinoma were 1.21, 2.33, 1.45, 2.05 and 1.52, respectively (Table 2). The score for each significant risk factor derived from the corresponding estimated regression coefficients is shown in Table 3. The total score was generated by the sum of the scores of each significant risk factor. The patients were divided into 9 groups according to the generated scores (0–8), which are shown in Figure 2A. Lung cancer patients can be separated into 3 groups on the basis of BM risk. Scores ranging from 0 to 4 indicated an incidence of 16.59% (2497/15,051); scores ranging from 5 to 6 indicated an incidence of 34.23% (191/558); and scores ranging from 7 to 8 indicated an incidence of 46.73% (50/107). The incidence in the 3 groups is shown in Figure 2B (*P* < .01).

**Table 1:** Demographic of the lung cancer patients.

Clinical subjects	Number of patients		*χ* ^2^		*P*-value
	With BM (N = 2738, 17.42%)	Without BM (N = 12978, 82.58%)			
Age, (yr)			6.70	.04	
18–45	205 (19.36)	854 (80.64)			
46–65	1767 (17.70)	8218 (82.30)			
≥66	766 (16.40)	3906 (83.60)			
Gender			12.68	<.001	
Female	1085 (18.84)	4673 (81.16)			
Male	1653 (16.61)	8301 (83.39)			
Unknown	0 (0)	4 (100)			
Marital status			1.28	.26	
Married	2685 (17.47)	12,684(82.53)			
Unmarried	47 (15.02)	266 (84.98)			
Unknown	6 (17.65)	28 (82.35)			
History of smoking			75.30	<.001	
None	1178 (20.73)	4504 (79.23)			
Yes	1459 (15.24)	8116 (84.76)			
Unknown	101 (22.00)	358 (78.00)			
Alcohol consumption			11.63	.001	
None	1702 (18.77)	7366 (81.23)			
Yes	780 (16.42)	3969 (83.58)			
Unknown	256 (13.48)	1643 (86.52)			
History of tumor			4.14	.04	
None	2672 (17.49)	12,608(82.51)			
Yes	50 (13.44)	322 (86.56)			
Unknown	16 (25.00)	48 (75.00)			
Family history of tumor			4.23	.04	
None	2125 (17.07)	10,327(82.93)			
Yes	596 (18.61)	2607 (81.39)			
Unknown	17 (27.87)	44 (72.13)			
KPS			159.97	<.001	
10–40	9 (34.61)	17 (65.38)			
50–70	347 (33.85)	728 (66.15)			
80–100	1464 (16.67)	7320 (83.33)			
Unknown	918 (15.74)	4913 (84.26)			
Primary lesions			15.70	<.001	
1	2597 (17.16)	12,533(82.84)			
>1	128 (23.75)	411 (76.25)			
Unknown	13 (27.66)	34 (72.34)			
Lymph node metastases			227.54	<.001	
None	1274 (13.64)	8065 (86.36)			
Yes	1403 (23.05)	4683 (76.95)			
Unknown	61 (20.96)	230 (79.04)			
Histological type			152.91	<.001	
Small-cell	261 (14.02)	1601 (85.98)			
Adenocarcinoma	1302 (18.72)	5654 (81.28)			
Squamous cell	293 (9.27)	2868 (90.73)			
Large cell	29 (13.30)	189 (86.70)			
Mixed	75 (16.09)	391 (83.91)			
Unknown	778 (25.48)	2275 (74.51)			

KPS = Karnofsky Performance Status.

**Table 2:** Univariate and multivariate regression analysis analyzing the demographic and related clinical characteristics for BM development in lung cancer.

Clinical subjects	Univariate		Multivariate	
	OR (95% CI)	*P*-value	OR (95% CI)	*P*-value
Age, (yr)				.27
18–45	1 (Reference)	1.00	1 (Reference)	1.00
46–65	0.90 (0.76–1.05)	.18	0.97 (0.76–1.23)	.78
≥66	0.82 (0.69–0.97)	.02	0.86 (0.66–1.12)	.26
Gender				
Female	1 (Reference)	1.00	1 (Reference)	1.00
Male	0.86 (0.79–0.93)	<.001	1.21 (1.02–1.44)	.03
Unknown	NA	NA	NA	NA
History of smoking				
None	1 (Reference)	1.00	1 (Reference)	1.00
Yes	1.46 (1.34–1.58)	<.001	1.15 (0.97–1.37)	.11
Unknown	NA	NA	NA	NA
Alcohol consumption				
None	1 (Reference)	1.00	1 (Reference)	1.00
Yes	1.18 (1.07–1.29)	.001	1.05 (0.89–1.23)	.59
Unknown	NA	NA	NA	NA
History of tumor				
None	1 (Reference)	1.00	1 (Reference)	1.00
Yes	0.73 (0.54–0.99)	.04	0.79 (0.51–1.24)	.32
Unknown	NA	NA	NA	NA
Family history of tumor				
None	1 (Reference)	1.00	1 (Reference)	1.00
Yes	1.11 (1.01–1.23)	.04	1.06 (0.92–1.23)	.43
Unknown	NA	NA	NA	NA
KPS				<.001
80–100	1 (Reference)	1.00	1 (Reference)	1.00
50–70	2.38 (2.07–2.74)	<.001	2.33 (1.93–2.82)	<.001
10–40	2.65 (1.18–5.95)	.02	1.77 (0.36–8.81)	.48
Unknown	NA	NA	NA	NA
Primary lesions				
1	1 (Reference)	1.00	1 (Reference)	1.00
>1	1.50 (1.23–1.84)	<.001	1.45 (1.02–2.06)	.04
Unknown	NA	NA	NA	NA
Lymph node metastases				
None	1 (Reference)	1.00	1 (Reference)	1.00
Yes	1.90 (1.74–2.06)	<.001	2.05 (1.80–2.33)	<.001
Unknown	NA	NA	NA	NA
Histological type				<.001
Small-cell	1 (Reference)	1.00	1 (Reference)	1.00
Adenocarcinoma	1.41 (1.22–1.63)	<.001	1.52 (1.26–1.83)	<.001
Squamous cell	0.63 (0.53–0.75)	<.001	0.70 (0.55–0.88)	.002
Large cell	0.94 (0.62–1.42)	.77	0.91 (0.51–1.61)	.74
Mixed	1.18 (0.89–1.56)	.25	1.19 (0.79–1.78)	.40
Unknown	NA	NA	NA	NA

KPS = Karnofsky Performance Status.

**Table 3:** Risk factors and assigned scores of bone metastasis in lung cancer.

Risk factors	Variables	Scores
Gender	Female	0
	Male	1
KPS	10–40	0
	50–70	3
	80–100	0
Primary lesions	1	0
	>1	1
Lymph node metastases	None	0
	Yes	2
Histological type	Non-adenocarcinoma	0
	Adenocarcinoma	1

The score for each significant risk factor was derived from the corresponding estimated regression coefficients (natural logarithm of the hazard ratio) of the multivariate model. The corresponding estimated regression coefficients were multiplied by 3 and rounded off to the nearest integer.

KPS = Karnofsky Performance Status.

## 4. Discussion

This study provided an understanding lung cancer with BM in the “real word,” without the selection pressures and biases that alter the make-up of a cancer population in a clinical trial. The results confirmed that BM is common in lung cancer patients. As reported, most BM patients present with BM at the time of lung cancer diagnosis. Only 30% of lung cancer patients developed BM later in the course of disease.^[4,10,22]^ Previous studies reported that 10% to 40% of patients with advanced lung cancer have BM.^[23–25]^ To our knowledge, the present study is the largest single-center study in China in which BM of lung cancer was studied, and it included 15,716 lung cancer patients. Male sex, a Karnofsky score of 50 to 70, multiple primary lesions, lymph node metastasis, and adenocarcinoma were proven to be significant independent risk factors for BM in patients with lung cancer. Data from the Surveillance, Epidemiology, and End Results database also revealed that male sex, lymph node metastasis, and adenocarcinoma were positively associated with BM development.^[25–27]^ A retrospective study including 407 lung cancer patients from Brazil also demonstrated that adenocarcinoma was an independent risk factor for BM in lung cancer patients.^[8]^ A similar trend between risk factors and BM was further confirmed in this study.

It is widely accepted that SREs significantly decrease the QOL of BM patients.^[28,29]^ Early identification of BM is critical for palliative treatment and SRE prevention. Lung cancer patients can benefit from the performance of BM screening, including: more comprehensive treatment opportunities for advanced lung cancer patients; timely bone-targeted therapy that can prevent the occurrence and development of SREs, thus preserving patient QOL; and individualized treatment plan generation.^[30–33]^ However, many factors hinder BM detection in patients with lung cancer. Currently, this diagnostic technique cannot satisfactorily meet the clinical needs of screening for BM in patients with lung cancer.^[34]^ The different imaging methods have different advantages and disadvantages. For example, positron emission tomography-computed tomography (PET-CT), which provides the optimum balance of accuracy and sensitivity, has been encouraged for patients with clear clinical symptoms, including bone pain, movement disorders, clinical pathological fractures, spinal cord compression, and spinal nerve compression. However, PET-CT is not a routine choice for screening for BM because of its high cost. Without risk classification, the performance of emission computed tomography (ECT), X-ray, and CT can increase the exposure of patients to radiation. Enhanced MRI scanning requires advanced equipment and skilled technicians, and the unnecessary application of MRI scanning increases the financial burden. Limited guidance has been given about screening for BM in patients with lung cancer.^[13]^ The lost opportunity for early diagnosis and bone lesion treatment could lead to suboptimal treatment decisions. Thus, an established scoring system is needed to guide individualized screening for patients with different BM risks.

According to the created scoring system, the incidence of BM in the different groups ranged from 16.59% to 46.73%. This finding can partly explain the different incidences of BM reported in previous studies. To guide clinical decision-making, we recommend the following approach for screening lung cancer patients with varying BM risks on the basis of their scores. For high-risk patients (scores of 7–8), we advise the use of high-sensitivity imaging modalities, such as bone scanning (ECT) or PET-CT, which are essential for the early detection of BM. For medium-risk patients (scores of 5–6), we suggest routine bone scanning (ECT) and recommend a follow-up review within 3 months. For low-risk patients (scores of 0–4), conventional imaging methods, such as X-ray or CT, should be used. In this way, monitoring and BM-related treatment could be coherent, and missed diagnoses of BM could be avoided.

There is strong evidence to support early intervention in patients with BM.^[33]^ Patients who experience 1 SRE are at increased risk of developing additional SREs, which may reduce the patient’s quality of life.^[10]^ Untimely intervention may result in unnecessary prolonged treatment exposure in patients, thus increasing potential adverse events. However, this risk should be minimized and weighed against the benefits of early intervention, such as preventing SREs and reducing the burden of SREs on patients and health care systems. In addition, SREs are associated with substantial health care resource utilization, including hospitalization and surgery. Thus, either prevention or delay of SREs will benefit both patients and health care systems.^[35]^ With the established scoring system, more patients with lung cancer BM can be identified in a timely manner, and the corresponding bone-targeting therapy can also prevent or delay SREs in patients.

Although we identified a series of independent risk factors for BM in patients with lung cancer and established a scoring system, several limitations remain. First, this study had a retrospective design, which may introduce selection bias and missing data, and some important clinical parameters were not included because of insufficient data. These factors may limit the accuracy and sensitivity of the established system and affect the generalizability of the results. Second, the present study was performed at a single center, and the limited sample size may not accurately reflect the true occurrence of BM in patients with lung cancer; thus, a multicenter prospective study is needed. Third, one of the key limitations of our study is the lack of external validation of the proposed model. The generalizability of our findings remains uncertain without validation in an independent cohort. Importantly, although the scoring system is promising, its applicability across different health care settings, such as resource-limited and high-resource environments, has not yet been tested. Further research is needed to evaluate its adaptability and predictive accuracy in diverse clinical contexts. To address this issue, we plan to undertake external validation in a subsequent study, where we will apply the scoring system to a separate dataset to assess its robustness and predictive accuracy in different populations.

## 5. Conclusion

A series of risk factors for BM in patients with lung cancer were identified, including male gender, a Karnofsky score of 50 to 70, multiple primary lesions, lymph node metastasis, and adenocarcinoma. A scoring system was established on the basis of the identified factors, and the risk of BM can be predicted. We present an auxiliary scoring system for lung cancer that can guide screening for BM and facilitate timely bone-targeted therapy.

## Author contributions

**Conceptualization:** Vladimir P. Chekhonin, Xu Guo, Chao Zhang.

**Data curation:** Weiqin Zhao, Haixiao Wu, Jun Wang, Jin Zhang.

**Formal analysis:** Weiqin Zhao, Jun Wang.

**Investigation:** Yile Lin, Jun Wang.

**Methodology:** Yile Lin, Haixiao Wu, Jun Wang.

**Project administration:** Andrey D. Kaprin, Xu Guo.

**Resources:** Haixiao Wu, Andrey D. Kaprin.

**Software:** Yile Lin, Haixiao Wu.

**Supervision:** Vladimir P. Chekhonin, Elmar R. Musaev, Jin Zhang, Chao Zhang.

**Validation:** Vladimir P. Chekhonin, Elmar R. Musaev, Jin Zhang, Chao Zhang.

**Visualization:** Elmar R. Musaev, Jin Zhang.

**Writing – original draft:** Weiqin Zhao, Vladimir P. Chekhonin, Elmar R. Musaev, Xu Guo, Chao Zhang.

**Writing – review & editing:** Andrey D. Kaprin, Xu Guo, Chao Zhang.
